# Prognostic Factors for Postoperative Visual Acuity in Patients with Rhinogenic Optic Neuropathy

**DOI:** 10.1155/2019/3417425

**Published:** 2019-10-03

**Authors:** Mitsuya Otsuka, Tatsuya Yunoki, Hironori Ozaki, Hirohiko Tachino, Hiromasa Takakura, Hideo Shojaku, Atsushi Hayashi

**Affiliations:** ^1^Department of Ophthalmology, Graduate School of Medicine and Pharmaceutical Sciences, University of Toyama, Toyama, Japan; ^2^Department of Otorhinolaryngolory, Graduate School of Medicine and Pharmaceutical Sciences, University of Toyama, Toyama, Japan

## Abstract

**Purpose:**

To examine the prognostic factors related to postoperative visual acuity in patients with rhinogenic optic neuropathy.

**Study Design:**

Retrospective observational study.

**Materials and Methods:**

We retrospectively studied the medical records for 15 eyes of 15 patients who underwent surgery for the treatment of rhinogenic optic neuropathy between 31 January 2010 and 30 April 2018 at Toyama University Hospital. The patient age, sex, preoperative and postoperative visual acuity, duration from visual deficit to surgery, use of steroids, type of rhinogenic optic neuropathy, and the part of sinus lesion were analyzed. We also examined postoperative visual acuity for patients whose preoperative visual acuity was less than light perception.

**Results:**

The type of optic neuropathy was sinusitis in 7 cases, mucocele in 5 cases, and pyocele in 3 cases. Visual acuity was improved in 9 cases and deteriorated or unchanged in 6 cases. Patients in the improvement group were significantly younger than those in the nonimprovement group (*p*=0.01). In univariate regression analysis, preoperative visual acuity and type of optic neuropathy significantly related to postoperative visual acuity. Finally, 4 of the 15 cases (27%) had preoperative visual acuity less than light perception, but there was an improvement in postoperative visual acuity in 2 of 4 cases (50%).

**Conclusions:**

Preoperative visual acuity is the predictive factor for postoperative visual acuity in patients with rhinogenic optic neuropathy, but even if the preoperative visual acuity is less than light perception, it can be improved by surgical treatment.

## 1. Introduction

Rhinogenic optic neuropathy is a condition in which sinus lesions affect the optic nerve, leading to vision impairment. Rhinogenic optic neuropathy is mainly divided into compressive optic neuropathy and optic neuritis. Pyocele and mucocele in the sphenoid sinus or ethmoid sinus are compressive optic neuropathy. They cause inflammation and pressure of the optic nerve and deteriorate visual acuity [1]. On the other hand, visual deterioration due to sinusitis caused by fungi and bacteria can also occur. The clinical symptoms of rhinogenic optic neuropathy are various and include visual disturbance, central scotoma, oculomotor palsy, and abducent palsy [2]. To treat the condition, the cyst is marsupialized using endoscopic sinus surgery in the sphenoid sinus or the ethmoid sinus, and communication with the nasal cavity is obtained [3–6].

However, the factors related to postoperative visual acuity in patients with rhinogenic optic nerve neuropathy are not clear.

The period between diagnosis and surgery has been considered as one visual prognostic factor. The shorter the period to surgery, the better the visual acuity [7]. However, other reports have found that there was no relationship between postoperative vision and either the duration of surgery or preoperative visual acuity [6]. The impact of postoperative steroid use on postoperative visual acuity is also unclear.

The purpose of this study was to investigate potential prognostic factors, i.e., age, sex, duration from visual deficit to surgery, preoperative visual acuity, use of steroids, type of rhinogenic optic neuropathy, and the part of sinus lesion, for their ability to predict postoperative visual acuity in patients treated for rhinogenic optic nerve neuropathy. We also specifically examined postoperative visual acuity in patients whose preoperative visual acuity was less than light perception.

## 2. Materials and Methods

We retrospectively studied the medical records for 15 eyes of 15 patients who underwent surgery for the treatment of rhinogenic optic neuropathy between 31 January 2010 and 30 April 2018 at Toyama University Hospital. Study approval was obtained from the institutional review board of Toyama University Hospital. All patients were placed under general anesthesia for endoscopic surgery by a hospital otolaryngologist. The wall of the mucocele or pyocele was removed using endoscopic sinus surgery techniques to release pressure on the optic nerve. To distinguish between mucocele and pyocele and fungal and bacterial infections, we relied on findings during surgery and culture results. There were no complications during the surgery.

In cases receiving postoperative steroid pulse therapy, either 1000 or 500 mg of methylprednisolone was administered for 3 days from the day after surgery as appropriate based on the patient's general condition. Short-term antibiotics were used for sinus surgery for sinusitis. A visual acuity test, fundus examination, computed tomography (CT), and magnetic resonance imaging (MRI) were performed before and after surgery, and it was confirmed that visual acuity had declined due to optic nerve disorder.

Classification of the type of rhinogenic optic neuropathy was divided into mucocele, pyocele, and sinusitis. In all of our cases with sinusitis, the condition appeared to have been caused by bacteria and fungi. Cases of tumor-induced optic neuropathy were excluded from the study. The visual acuity was calculated as the logarithm of the minimum angle of resolution (logMAR) value as described in a previous report [8]; the visual acuity in our patients was 2.8 for those with light perception and 2.9 for those without light perception.

The patient's age, sex, preoperative and postoperative visual acuity and duration from visual deficit to surgery, use of steroids, type of rhinogenic optic neuropathy, and the part of sinus lesion were analyzed. We also examined postoperative visual acuity for patients with preoperative visual acuity less than light perception. All statistical analyses were performed using the logMAR values with IBM® SPSS ®Statics 21 (Loyola University Chicago, Chicago, IL). *p* values less than 0.05 were defined as statistically significant. Univariate regression was used to assess correlations between postoperative visual acuity and clinical parameters.

## 3. Results and Discussion

### 3.1. Results

The clinical characteristics of the 15 cases are shown in Table 1. The mean age was 67.8 ± 16.4 years. The mean duration from visual deficit to surgery was 7.6 ± 5.4 days. One case had a large excavation of the optic nerve head, but the other cases had normal fundi. The type of optic neuropathy was sinusitis in 7 cases, mucocele in 5 cases, and pyocele in 3 cases. Three eyes had oculomotor nerve palsy. There were 6 cases with paranasal surgery history, and there were 11 cases in which postoperative steroid pulse was performed. Eight cases had lesions in the sphenoid sinus and 9 cases had lesions in the ethmoid sinus.

**Figure 1:**
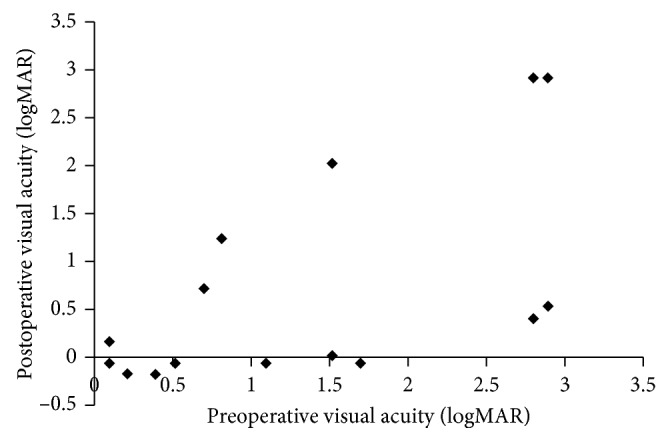
Preoperative and postoperative visual acuities.

The mean preoperative visual acuity (logMAR) was 1.34 ± 1.03, and the mean postoperative visual acuity (logMAR) was 0.674 ± 1.05 (*p*=0.018) (Figure 1). When converted to decimal visual acuity, it improved from 0.05 to 0.21. Visual acuity was improved in 9 cases and was deteriorated or unchanged in 6 cases (Table 2). Visual acuity was deteriorated in 4 cases (aspergillosis 3 cases and pyocele 1 case). The age was significantly younger in the improvement group than in the nonimprovement group (*p*=0.01), but sex, preoperative visual acuity, presence or absence of steroid pulse application, and type of optic neuropathy were not significantly different between the two groups. Although there was no significant difference with respect to duration from visual deficit to surgery, shorter duration tended to be more common in the visual acuity improvement group (*p*=0.0504).

Postoperative visual acuity is shown on the vertical axis and preoperative visual acuity on the horizontal axis. The visual acuity was calculated by the logarithm of the minimum angle of resolution (logMAR) value.

In univariate regression analysis for postoperative visual acuity, the factors significantly related to postoperative visual acuity were preoperative visual acuity and pyocele (Table 3). Four of the 15 cases (27%) had no light perception or light perception preoperatively, but 2 (50%) of these cases showed improvement in visual acuity postoperatively. The visual acuity of the two eyes improved to 0.5 and 0.4 (logMAR), respectively (Table 4).

### 3.2. Discussion

The usefulness of endoscopic paranasal sinus surgery for rhinogenic optic neuropathy has been reported in several reports [4, 6]. Nakaya et al. reported that visual acuity improved in 31/38 eyes of patients with paranasal mucocele treated with endoscopic sinus surgery [6]. Lee et al. reported on 15 eyes (6 in cases with sinusitis, 4 in cases with mucocele, and 5 in cases with aspergillosis) in patients undergoing endoscopic surgery for sphenoid disease and found that visual acuity was improved in 9/15 eyes [4]. Although the prognostic factors for rhinogenic optic neuropathy have been studied in the past, these analyses have been limited due to the small number of cases. In the present analysis, we examined potential prognostic factors, such as the age, sex, type of neuropathy, presence or absence of steroid pulse application, duration from visual deficit to surgery, preoperative visual acuity, and the part of sinus lesion. Our results showed that younger age was significantly associated with an improvement in visual acuity and preoperative visual acuity, and type of optic neuropathy significantly was related to postoperative visual acuity.

Steroid pulse therapy is sometimes used for visual acuity improvement after surgery for rhinogenic optic neuropathy, and in our study, steroid pulse therapy was conducted in 73% of cases. However, we did not observe any significant differences in the prognosis of visual acuity based on the presence or absence of steroid pulse therapy. Two previous studies similarly reported that steroid pulse did not affect visual acuity [5, 6]. Therefore, depending on the general condition of the patient, we may not necessarily employ steroid pulse therapy. Nonetheless, further analysis will be needed in a large number of cases before reaching any definitive conclusions.

There are several reports on the duration from visual deficit to surgery. Yoon et al. reported that the visual improvement was better if the time from the occurrence of visual acuity reduction to surgery was less than 2 weeks for sphenoid sinus disease in 14 eyes (5 cases with mucocele, 2 cases with pyocele, and 1 case each with meningioma, pituitary adenoma, adenoid cystic carcinoma, sphenoiditis, aspergilloma, invasive aspergillosis, and mucormycosis) [5]. Yumoto et al. reported that prominent visual acuity improvement was obtained if the surgery could be performed within 24 hours, in sphenoethmoid mucoceles [7]. Moriyama et al. reported on the surgical treatment of cases of mucocele in the sphenoid and ethmoid sinus and found that the extent of visual acuity improvement depended on the time from disease onset until surgery [9]. On the other hand, Nakaya et al. reported that the period of vision impairment was not significantly related to complete postoperative recovery of vision [6]. In our study, the number of days from vision reduction to surgery tended to be shorter in the vision improvement group. A relatively large number of reports indicate that early operation is an important factor for visual acuity improvement, and if possible, we should diagnose early and consult to otolaryngology.

It has been reported that there is no relation between age and visual acuity improvement in patients with surgically treated rhinogenic optic neuropathy [6]. In our present study, age was significantly younger in the visual acuity improvement group. Because the duration from visual deficit to surgery tended to be short in the visual acuity improvement group, we considered that surgery should be performed early before the visual disturbance becomes strong.

It has been reported that there is no relation between the part of sinus lesion and visual acuity improvement in patients with surgically treated rhinogenic optic neuropathy [5, 6]. In this study as well, the postoperative visual acuity was not significantly deteriorated due to the difference in sinus lesions.

It has been reported that there is relation between type of optic neuropathy and visual acuity improvement in patients with surgically treated sphenoid sinus disease [5]. In this report, fungi and sphenoid sinusitis have poor visual acuity, and direct optic nerve infiltration because infection is considered to cause poor recovery. In our report, pyocele was a significant factor in poor postoperative visual acuity in univariate regression analysis. This suggests that pyocele may have poor postoperative visual prognosis due to pressure on the optic nerve and infiltration into the optic nerve due to infection compared to mucocele. Visual acuity was deteriorated in the 4 cases (aspergillosis 3 cases and pyocele 1 case) despite surgical treatment, antibacterial drugs, and steroid in our study. In all four cases, infection is involved. It is thought that optic nerve infiltration occurred and the postoperative visual acuity was poor.

Finally, we examined cases with preoperative visual acuity less than light perception. There have been several reports on the results of surgery for rhinogenic optic neuropathy in patients with visual acuity less than light perception [6, 10]. In one report, no recovery was observed in 2 cases of rhinogenic optic neuropathy without light perception (the intervals between the onset of visual disturbance and surgery were 4 days and 16 days) [6]. Hiratsuka et al. performed surgical treatment (sphenoidotomy) followed by treatment with systemic steroids and antibiotics for rhinogenic optic neuropathy caused by a mucocele in a patient with preoperative bilateral light perception, but the light perception disappeared even though the surgery was performed within 24 hours of onset [10]. Preoperative visual acuity less than light perception is reported to be a poor prognosis, but there are also reports in which visual acuity recovered after surgery [3, 6, 7]. Nakaya et al. reported that the visual acuity was improved in 5 of their 6 patients with preoperative visual acuity of light perception [6]. Another report showed that the number of days from surgery was 1–4 in 5 cases with visual acuity improvement, versus 3 months in the case with no improvement [3]. Yumoto et al. reported that 3 out of 5 cases without light perception showed some improvements in visual acuity by the surgery within 24 hours after onset of visual loss [7]. Therefore, because early surgery has generally been performed in the reported cases with an improvement in visual acuity, early diagnosis and treatment are recommended. In our study, visual acuity improvement was seen in 1 of 2 cases with no light perception. In the cases where improvement was obtained, surgical treatment could be performed on the same day when vision deterioration occurred. Since there is a possibility of improvement even if there is no light perception, such cases should be diagnosed and surgically treated as soon as possible.

This research has a limitation. The limitation of this study is that it is a retrospective study and had a small number of 15 cases. In this study, duration from visual deficit to surgery was not significantly associated with visual improvement, but a statistically significant difference may be detected by examining more cases.

## 4. Conclusions

In conclusion, we examined factors related to the postoperative visual acuity of patients treated surgically for rhinogenic optic neuropathy. Our results showed that preoperative visual acuity was the factor most predictive of a postoperative improvement in visual acuity. However, an improvement in visual acuity could be obtained even in cases without light perception. It is thus important that rhinogenic optic neuropathy be diagnosed as soon as possible and surgically treated by an otolaryngologist.

## Figures and Tables

**Table 1:** Clinical characteristics of the 15 cases.

Mean age	67.8 ± 16.4
Male : Female	8 : 7
Type of optic neuropathy	
Sinusitis	7/15 (47%)
Bacteria : fungi	3 : 4
Mucocele	5/15 (33%)
Pyocele	3/15 (20%)
Fundus findings	1 eye: large excavation of optic nerve head
	14 eyes: normal fundus
Sinus lesion	
Sphenoid	8 (53%)
Ethmoid	9 (60%)
Oculomotor nerve palsy	3/15 (20%)
Sinus surgery history	6/15 (40%)
Steroid pulse	11/15 (73%)
Duration from visual deficit to surgery	7.6 ± 5.4 (days)
Preoperative mean visual acuity (logMAR)	1.34 ± 1.03

MAR: minimum angle of resolution.

**Table 2:** Comparison of the ratio of visual prognostic factors in the visual acuity improvement group and the nonimprovement group by surgery.

	Visual acuity improvement *N* = 9	Visual acuity nonimprovement *N* = 6	*p* value
Age	59.8 ± 15.9	79.8 ± 7.3	0.01^*∗*^

Sex			
Male	4	4	0.61
Female	5	2	

Duration from visual deficit to surgery (days)	5.7 ± 2.7	12 ± 7.1	0.05

Steroid pulse			
+	8	3	0.24
−	1	3	

Preoperative visual acuity (logMAR)	1.25 ± 1.06	1.47 ± 1.06	0.71

Type of optic neuropathy			
Mucocele	4	1	0.58
Pyocele	1	2	0.52
Sinusitis	4	3	1

Sinus lesion			
Sphenoid	5	3	1
Ethmoid	5	4	1

MAR: minimum angle of resolution, ^*∗*^*p* < 0.05, Student's *t*-test, Fisher exact test.

**Table 3:** Univariate regression on postoperative visual acuity.

	Coefficient	*p* value
Age	0.027	0.114
Sex	0.466	0.428
Steroid pulse	0.241	0.719
Duration from visual deficit to surgery	−0.021	0.701
Preoperative visual acuity (logMAR)	0.613	0.018^*∗*^
Type of optic neuropathy		
Sinusitis	−0.211	0.722
Mucocele	−0.747	0.222
Pyocele	1.366	0.047^*∗*^
Sinus lesion		
Sphenoid	−0.874	0.12
Ethmoid	0.704	0.23

MAR minimum angle of resolution, ^*∗*^*p* < 0.05, univariate linear regression analysis.

**Table 4:** Postoperative visual acuity of patients with preoperative visual acuity less than light perception.

Sex	Age (years)	Pre-visual acuity	Post-visual acuity	Type of optic neuropathy	Duration from visual deficit to surgery (days)	Steroid pulse	Sinus surgery history
Female	64	NLP	0.3	Mucocele	0	Yes	Yes
Male	85	LP	NLP	Sinusitis	10	Yes	No
Male	74	LP	0.4	Pyocele	4	Yes	Yes
Male	76	NLP	NLP	Pyocele	4	Yes	Yes

## Data Availability

The clinical data used to support the findings of this study are included within the article.
